# Mental health of postgraduate trainees in primary care: a cross-sectional study

**DOI:** 10.1186/s12875-020-01199-6

**Published:** 2020-06-27

**Authors:** Till J. Bugaj, Katja Krug, Annalena Rentschler, Christoph Nikendei, Joachim Szecsenyi, Simon Schwill

**Affiliations:** 1 Department of General Internal Medicine and Psychosomatics, University Hospital Heidelberg, Heidelberg, Germany; 2 Department of General Practice and Health Services Research, University Hospital Heidelberg, Marsilius Arkaden, INF 130.3, Turm West, 69120 Heidelberg, Germany

**Keywords:** Mental health, Depression, Stress, Burnout, Primary care, General practice, Postgraduate education, Residency, Vocational training

## Abstract

**Background:**

General Practitioners (GPs) are increasingly affected by stress-related complaints and burnout. Although many studies have addressed this issue, little is known about the stress burden and burnout rates of postgraduate trainees specialising in General Practice (GP). This cross-sectional study was performed to explore the prevalence and risk of depression, stress and burnout in a large cohort of GP trainees.

**Methods:**

All GP trainees enrolled in the postgraduate training programme KWBW Verbundweiterbildung^*plus*^© in southwest Germany were invited to participate. A paper-based survey for the purpose of psychosocial screening was used: Prevalence of depression, perceived stress and burnout were measured with the depression module of the Patient Health Questionnaire (PHQ-9), Perceived Stress Questionnaire (PSQ-20) and Maslach Burnout Inventory (MBI). Additionally, linear regression models were used to analyse the association between sociodemographic characteristics and mental health scales.

**Results:**

*N* = 211 GP trainees participated in this study (response rate 95%). 75.3% (*n* = 159) of the participants were female and median age was 34 (IQR 32; 39). GP trainees had a mean PHQ-9 sum score of 5.4 (SD 3.4). Almost 11% (*n* = 23) reported symptoms of a moderate or moderately severe depression. PSQ-20 revealed moderate level of distress, whereas 20.8% (*n* = 42) showed a high level of perceived stress with a sum-score higher than .59. GP trainees showed moderate rates of burnout and only 2.5% (*n* = 5) scored high in all three dimensions of the MBI score. GP trainees showed increased levels of depression, perceived stress and burnout when compared with age-matched general population. Being a woman led to a higher PHQ-9 sum score (*p* < .05). Higher age was associated with less depersonalisation in the MBI (p < .05).

**Conclusions:**

The results of our study suggest that GP trainees considerably suffer from stress. Some GP trainees were even affected by depression or burnout. To detect and support colleagues at risk, trainees should be supported by early preventive measures such as anti-stress or resilience trainings and mentoring during their training. Prospective longitudinal studies are needed to understand the character and the course of the stress burden among GP trainees.

## Background

General practitioners (GPs) often are not satisfied with their working conditions [1]. In addition, GPs are burdened for many different reasons, e.g. the growing workload, patients with complex psychosocial and medical problems as well as the need to keep up to date with many different diseases and bureaucratic changes [2, 3], and are therefore quite often affected by stress-related complaints. In fact, GPs seem to be particularly affected by physician burnout [4], although absolute levels of burnout vary from study to study [5–7]. At the same time the international literature about the prevalence of stress, burnout and depression among physicians in training (= vocational trainees /residents) is alerting [8–10]. Only recently researchers from Mayo Clinic demonstrated that symptoms of burnout occurred in nearly half of US resident physicians, with a wide range in prevalence by clinical specialty [11]. In addition, 14% of the residents in Dyrbyes study regretted their career choice [11]. Those residents suffering from burnout had more than a threefold increase in odds of regretting their decision to become a physician, which - not only in view of the high study costs and the years of training – is a rather alarming sign. However, little is known about the work strain and burnout rates of postgraduate trainees specialising in General Practice (GP).

The aim of this cross-sectional study was to explore the risk of GP trainees to suffer from depression, stress and burnout and thereby quantify the level of burden among German postgraduate trainees specialising in GP. Therefore all GP trainees enrolled in the postgraduate training programme KWBW Verbundweiterbildung^*plus*^© in southwest Germany were invited to participate in an anonymous paper-based survey for the purpose of psychosocial screening.

We hypothesised that GP trainees would considerably suffer from depression, stress and burnout and might be similar burdened as their more experienced GP colleagues.

## Methods

### Study design

In this cross-sectional study we investigated the psychological measures of GP trainees prior to a seminar on self-care and coping with stress.

### Setting

GP specialist training in Germany takes about 5 years and requires hospital and ambulatory rotations. To increase the attractiveness of GP in face of the looming shortage of GPs in Germany, the first German training programme for GP was founded in 2008 in Baden-Württemberg in the southwest of Germany: Today, the KWBW Verbundweiterbildung^plus^© offers a curricular seminar programme, a structured mentoring programme and regional clinical rotations all over Baden-Württemberg for GP trainees [12–14]. The seminar programme offers education with emphasis on case-based seminars to enable a constructive alignment with everyday work of the clinical rotation. Mental health and self-care as a physician is also part of the 5-year core curriculum, while the catalogue of requirements for GPs (“Logbuch”) does not include any reference to individual psychosocial health of the GPs [15].

Participation in the seminar programme is voluntarily and financial resources are low which is why each participant can be offered only up to 36 h of seminar programme per year. Every participant of the programme can visit four one-day training session and one two-day training session per year. The data presented was generated during the two-day training sessions in 2018. Schedule of the 2018 two-day seminar included a 270-min session on self-care and coping with stress tailored to the needs of GP trainees during the second day of the course. In total, *n* = 9 two-day-courses took place in 2018 with a total maximum capacity of *n* = 250 participants.

### Sample

Participation in one of the two-day courses (maximum capacity of n = 250, see above) was offered to all GP trainees enrolled into the programme in 2018. All participants of the seminar were then invited to take part in the study. GP trainees integrated in planning and/or performing the study were excluded.

### Assessment of psychometric characteristics

Before the start of the seminar on self-care and coping with stress as a GP trainee study participants were asked to fill in a questionnaire for the purpose of psychosocial screening. The following instruments were used:

### Patient health Questionnaire-9

Depressive symptoms were measured using the validated German version of the Patient Health Questionnaire-9 (PHQ-9), a self-administered version of the PRIME-MD diagnostic instrument. For the PHQ-9 each of the nine DSM-IV criteria is scored as “0” (not at all) to “3” (nearly every day), so that the total score ranges from 0 to 27.

The instrument can not only be utilized to make criteria-based diagnoses of depressive disorders, it is also a well validated and reliable research tool [16]. Scores of 10 or greater, 15 or greater and 20 or greater correspond to moderate, moderately severe and severe depression respectively [17]. A score ≥ 10 on the PHQ-9 already shows a sensitivity of 93% and a specificity of 88% for the diagnosis of major depressive disorder [16] .

### Perceived stress Questionnaire-20

The Perceived Stress Questionnaire (PSQ) is a validated instrument for measuring perceived stress independent of a specific context [18]. In this study, the German short version of the PSQ with only 20 items (PSQ-20) was utilized [19]. Items on the PSQ-20 are rated on a 4-point Likert scale. The PSQ-20 entails four subscales; *Worries*, *Tension* and *Loss of Joy* (all of which measure the individual’s internal stress reactions), as well as *Demands* (which represents the individual’s general perception of external stressors). PSQ-20 sum scores range to values 0–100 after linear transformation [19]. We used the PSQ-20 cut-offs recently defined by Kocalevent et al. [20]:

- PSQ-20 sum ≤0.45: regular
- PSQ-20 sum > 0.45 - ≤0.59 moderate level of perceived stress
- PSQ-20 sum ≥0.60 high level of perceived stress

### Maslach burnout inventory

The Maslach Burnout Inventory (MBI) can be considered as international gold standard for the measurement of burnout [21]. We used the validated German version (MBI-D) of the Maslach Burnout Inventory-Human Services Survey (MBI-HSS) by Enzmann and Kleiber [22] without questions 23–25 (initial fourth dimension of “involvement” – redundant for statistical reasons) and without an additional intensity evaluation (originally there were additional subscales in the MBI to further evaluate each item in terms of its intensity). This approach left us with 22 questions, divided into three scales: Emotional exhaustion (EE), depersonalisation (DP, which was renamed “cynicism” when the MBI General Survey was introduced), and personal accomplishment (PA, renamed “professional efficacy” with the introduction of the MBI General Survey). The items of the MBI-HSS are scaled from 0 (never) to 6 (always) and must theoretically be considered as ordinally scaled [23], although the handbook of Maslach et al. [24] does not contain total sum scores but recommends to utilize three summed subscales.

The higher the values in the scales EE and DP and the lower the values in the scale PA are, the greater is the risk of burnout. In accordance with the EGPRN study [7], the following cut-offs were used for our study:

- MBI-EE: low burnout ≤13, average burnout 14–26, high burnout ≥27
- MBI-DP: low burnout ≤5, average burnout 6–9, high burnout ≥10
- MBI-PA: high burnout ≤33, average burnout 34–39, low burnout ≥40 (*Cave*: inverse scale)

### Statistical data analysis

Data were analysed using the software package IBM SPSS Statistics 22. Descriptive statistics, such as means, standard deviations and frequencies were calculated. Pearson’s correlation coefficient was used to describe interdependence between mental health scales. Linear regression models analysed the association between sociodemographic characteristics (age, gender, year of training, rotation) and mental health scales. Variables were included in one block (missing listwise).

## Results

### Sample

On January 1st in 2018 *n* = 401 were registered in the programme and were offered attendance in the two-day seminar. A total of *n* = 224 (55.9%) attended the seminars and could thus have taken part in this study. *N* = 3 GP trainees were excluded because they were involved into the planning of the study. Out of *n* = 221 eligible participants, a total of *n* = 211 GP trainees finished the required questionnaires for participation (response rate: 95.5%), with a maximum of *n* = 210 valid returns per instrument/dimension.

Table 1 depicts participants’ baseline characteristics. The majority of the participants were female. Since there were some career changers in the programme who had been active as physicians in other disciplines before, there is a high variance in age. The mean age was 36.2 years (SD 6.6). More than two thirds of the participants were in year 4 or 5 of postgraduate training, i.e. in the final stage of their trainings where they actually work in GP practices and not in hospitals anymore.

**Table 1:** Sociodemographic data of participating GP trainees (*n* = 211)

	n (%)
**Gender**	
female	159 (75.3%)
male	51 (24.2%)
unknown	1 (0.5%)
**Age in years** Md (Q1; Q3)	
34 (32; 39)	
≤ 25	0 (0.0%)
26–30	27 (12.8%)
31–35	92 (43.6%)
36–40	43 (20.5%)
41–45	18 (8.5%)
46–50	17 (8.0%)
> 50	8 (3.8%)
unknown	6 (2.8%)
**Year of training**	
1st	19 (9.0%)
2nd	16 (7.6%)
3rd	27 (12.8%)
4th	78 (36.8%)
5th	65 (31.0%)
unknown	6 (2.8%)
**Rotation**	
outpatient/practice	116 (55.0%)
hospital	29 (13.7%)
unknown	66 (31.3%)

***Note.****GP* General Practice, *Md* Median, *Q* quartile

### Psychosocial burden of GP trainees

Table 2 shows the scores of the PHQ-9, PSQ-20 and MBI instruments.

**Table 2:** Depression (PHQ-9), stress (PSQ-20) and burnout (MBI) among postgraduate trainees in GP

	n (%)	M (SD)	Md (IQR)
Level of depression (PHQ-9)			
PHQ-9 sum score (0–27)	208 (98.6)	5.4 (3.4)	5 (3; 7)
None (0)	3 (1.4)		
Minimal [1–4]	89 (42.2)		
Mild [5–9]	93 (44.1)		
Moderate [10–14]	20 (9.5)		
Moderately severe [15–19]	3 (1.4)		
Severe [20–27]	–		
Major depression (%)	8 (3.8)		
Other depression (%)	9 (4.3)		
Level of perceived stress (PSQ-20)			
worries	208 (98.6)	0.3 (0.2)	0.3 (0.1; 0.4)
tension	207 (98.1)	0.4 (0.2)	0.5 (0.3; 0.6)
joy	207 (98.1)	0.6 (0.2)	0.6 (0.5; 0.8)
demands	207 (98.1)	0.5 (0.2)	0.5 (0.4; 0.7)
sum score	202 (95.7)	0.4 (0.2)	0.4 (0.3; 0.6)
Level of feeling burnout (MBI)			
MBI-EE	204 (96.7)	22.3 (9.8)	21 (15; 29)
MBI-DP	210 (99.5)	8.1 (5.2)	7 (4; 11)
MBI-PA	206 (97.6)	39.7 (5.6)	41 (36; 44)

**Note.***PHQ-9* Patient Health Questionnaire-9, *PSQ-20* Perceived Stress Questionnaire-20, *MBI* Maslach Burnout Inventory, *MBI-EE* Dimension of the MBI “Emotional Exhaustion”. *MBI-DP* Dimension of the MBI “Depersonalisation”, *MBI-PA* Dimension of the MBI “Personal Accomplishment”, *M* mean, *SD* standard deviation, *Md* median, *IQR* interquartile range

### PHQ-9

**Fig. 1:**
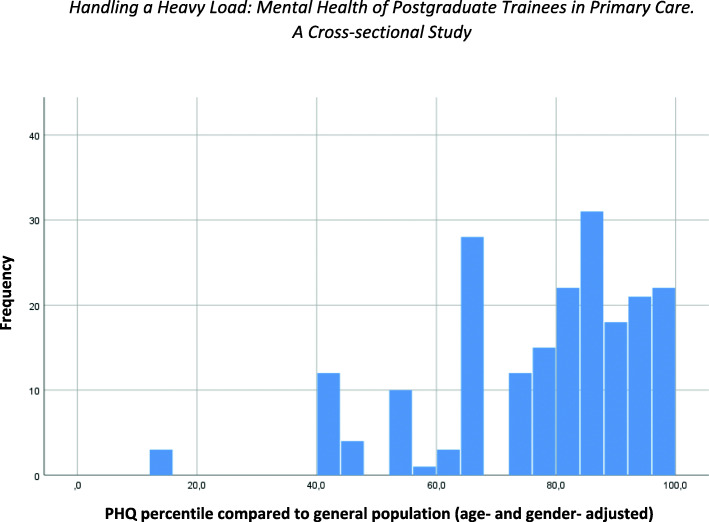
Level of depression in GP-trainees: PHQ-9 percentile in comparison with the general population

Regarding the PHQ-9 the participants had a mean score of 5.4 (SD 3.4) which corresponds with mild depressive symptoms, whereas a total of 10 or above is suggestive of the presence of depression. Almost 11% of the GP trainees showed symptoms of a moderate or even moderately to severe depression. Mean PHQ-9 in comparison to the general population is shown in Fig. 1.

### PSQ-20

The PSQ-20 results suggest a moderate level of distress – in accordance to Kocalevent [20] with sum scores ranging between .46 and .59 – in *n* = 40 (19.8%) participants. A total of *n* = 42 (20.8%) of the trainees showed a high level of perceived stress with sum scores higher than .59 in the PSQ-20.

### MBI

GP trainees showed moderate symptoms in terms of burnout with *n* = 5 (2.5%) scoring high in all three dimensions of the MBI score, whereas *n* = 96 participants (47.1%) reached no high score in any dimension. The distribution in relation to the individual burnout categories per dimension is shown below:

- MBI-EE (*n* = 203): low burnout *n* = 41 (20.2%), average burnout *n* = 94 (46.3%), high burnout *n* = 68 (33.5%).
- MBI-DP (*n* = 210): low burnout n = 68 (32.4%), average burnout n = 68 (32.4%), high burnout *n* = 74 (35.2%)
- MBI-PA (*n* = 206): low burnout *n* = 117 (56.8%), average burnout *n* = 59 (28.6%), high burnout *n* = 30 (14.6%)

### Pearson’s correlations

PHQ-9 sum score highly correlated with PSQ-20 sum score (*r* = .73). PSQ-20 sum score highly correlated with MBI-EE (*r* = .64). Pearson’s correlations of the tools and their subdimensions are shown in Table 3.

**Table 3:** Pearson’s correlation of the PHQ-9 sum score, PHQ-9 percentile, PSQ-20 subscales and the MBI dimensions

		[a]	[b]	[c]	[d]	[e]	[f]	[g]	[h]	[i]^**d**^
PHQ-9 sum score (0–27)	*r*	.83^a^	.72^a^	.67^a^	.64^a^	.51^a^	.73^a^	.53^a^	.38^a^	.23^a^
	n	202	205	204	204	204	199	201	204	208
PHQ-9: percentile [a] ^c^	*r*		.66^a^	.65^a^	.62^a^	.50^a^	.70^a^	.52^a^	.38^a^	.26^a^
	n		200	199	199	199	194	195	198	202
PSQ-20 scale: worries (0–1) [b]	*r*			.73^a^	.72^a^	.66^a^	.88^a^	.57^a^	.36^a^	.19^a^
	n			206	206	206	202	201	203	207
PSQ-20 scale: tension (0–1) [c]	*r*				.76^a^	.72^a^	.92^a^	.57^a^	.43^a^	.24^a^
	n				205	205	202	200	202	206
PSQ-20 scale: joy (0–1) [d]	*r*					.56^a^	.87^a^	.60^a^	.56^a^	.30^a^
	n					205	202	200	202	206
PSQ-20 scale: demands(0–1) [e]	*r*						.84^a^	.51^a^	.31^a^	.15^b^
	n						202	201	203	206
PSQ-20 scale: sum score (0–1) [f]	*r*							.64^a^	.47^a^	.27^a^
	n							196	198	201
MBI-EE [g]	*r*								.34^a^	.54^a^
	n								200	203
MBI –PA (reverse) [h]	*r*									.32^a^
	n									205

***Note.****r =* Pearson’s correlation coefficient: .**0–.20:** no correlation **.20–.40:** mild correlation **.40–.60:** moderate correlation **>.60:** high correlation **>.80:** strong correlation ^**a**^**.** Level of significance: 0.01 (2-sided).^b^. Level of significance: 0.05 (2-sided). ^c^compared to the general population (age- and gender-standardised). ^d^ [i] Maslach Burnout Inventory Depersonalisation Scale (MBI-DP). PHQ-9: Patient Health Questionnaire-9. PSQ-20: Perceived Stress Questionnaire-20. MBI: MBI-EE: Dimension of the Maslach Burnout Inventory “Emotional Exhaustion”. MBI-PA: Dimension of the Maslach Burnout Inventory “Personal Accomplishment

### Association of sociodemographic characteristics and mental health

Linear regression models showed no association between sociodemographic characteristics (age, gender, year of training, rotation) and PSQ-20 sum score, MBI-EE or MBI-PA. Considering all available sociodemographic factors, the MBI-DP was negatively associated with age (higher age is associated with less depersonalisation <.05). Being a woman led to a higher PHQ-9 sum score (*p* < .05). Other associations with the PHQ-9 could not be found.

Due to substantial missing values in the variable “rotation”, linear regression models were also calculated without this variable. The second regression analysis showed the same results and additionally revealed, a negative association between age and MBI-EE (higher age is associated with less emotional exhaustion, p < .05).

## Discussion

The present study is, to the best of our knowledge, the first to explore the psychosocial burden of GP trainees in three different domains; testing for depression, stress burden and burnout. More than one GP trainee out of ten presented with symptoms of moderate to severe depression. One out of five GP trainees showed a high level of perceived stress (PSQ-20). Few GP trainees showed moderate rates of burnout scoring high in all three dimensions of the MBI score, with the older participants in particular reporting less emotional exhaustion and less depersonalisation.

### Mental health of GP trainees in comparison to general population

In our own sample the mean PHQ-9 score was rather high, especially when compared to general population (see Fig. 1). Additionally, the GP trainees had a higher level of perceived stress than the general population: According to Fliege et al. healthy adults show PSQ-scores of 0.33 (SD 0.17) [25]. Kocalevent et al. showed in a more recent study [20] in a representative sample of the German general population (*n* = 2.552; households and target persons were selected at random) a mean PSQ-score of 0.3 (SD 0.15). In our own sample we found a mean PSQ-20 score of 0.4 (SD 0.18), while *n* = 40 (19.8%) of GP trainees showed PSQ-20 sum scores ranging between 0.45 and 0.59 being indicative of a moderate level of distress. A total of *n* = 42 (20.8%) of the trainees even showed a high level of perceived stress with sum scores higher than 0.59. It is worth noticing that Fliege et al. used the original 30-items version to establish the aforementioned PSQ-mean values [25]. However, the shorter version (PSQ-20), which we used for this study, was validated after the reduction from 30 to 20 items [19] and the results of both instruments can be compared after linear transformation of the resulting total score between 0 and 1.

In contrast, it is difficult to compare the mean MBI-scores of our study sample to the general population, as we were using the MBI-HSS, which was specially developed to assess burnout in the human services and *not* in general population [24]. To detect signs of burnout in other organisational contexts, the MBI-GS was developed [26], which is difficult to compare with the MBI-HSS due to the different number of items. We looked at a study on the experience of burnout in healthcare professionals from the private hospitals in Delhi, India [27], as the authors used the MBI-HSS also for their support staff (i.e. heterogeneous group of staff not working as physicians or nurses, e.g. security, pharmacy, front office, housekeeping, etc.). This subgroup can therefore be considered a “broader” population, although we are aware of the existence of possible regional characteristics. The support staff showed a mean MBI-EE score of 16.62 (SD 3.78), a mean MBI-DP score of 7.82 (SD 1.59) and a mean MBI-PA score of 34.69 (SD 3.67). Thus our study sample shows higher burden in the burnout-dimensions of emotional exhaustion and depersonalisation and only seems to be less burdened in terms of personal accomplishment.

### Mental health of GP trainees in comparison to other physicians

As far as we know, there is no study of high quality using the PHQ-9 in a bigger sample of fully-trained GPs. However, Schwenk et al. screened randomly selected practicing physicians, i.e. those being professionally active and not in training, from Michigan for depression using the PHQ-9 [28]. They received a total of *n* = 1.152 usable responses with those active in primary care being the biggest subgroup. Of this study population 11.3% scored positive for moderate to severe depression. In our sample of GP trainees an almost identical share of 10.9% showed signs of moderate to severe depression.

We are not aware of a study using the PSQ in a bigger sample of (fully-trained) GPs. Bernburg et al. [29] used the PSQ to measure perceived stress in residents from various specialties in German hospitals with an average work experience of 4 years (SD 2 years). Bernburg’s study participants displayed a mean score of 0.48 (SD 0.18) which is a little higher than our mean PSQ-20 score, while the total prevalence of perceived stress at a moderate level was 39.5% and at a high level 17.1%, which is comparable to our own data.

A number of studies [5–7] have used the MBI to describe burnout in GPs. Due to its big sample size and its multi-centered approach the so-called EGPRN study is especially well-known [7]. In the EGPRN study almost 3500 questionnaires featuring the MBI-HSS were distributed in 12 European countries with a response rate of 41%. Participants of the EGPRN study had a mean time of 19.2 years since graduation (SD 8.5). The reported mean MBI-EE score in the EGPRN study was 24 (SD 16) as opposed to a slightly lower mean score of 22.3 (SD 9.8) in our own study sample. 33.5% of GP trainees in our study scored high in the MBI-EE dimension (vs. 43% of participants in the EGPRN study). In terms of depersonalisation (MBI-DP) fully-trained GPs showed a mean score of 7 (SD 7). In this dimension the German GP trainees from our own study seemed more burdened than their European colleagues – they had a mean MBI-DP score of 8.1 (SD 5.2). 35.2% of our participants scored high in this dimension (vs. 35.3% in the EGPRN study). The GP trainees in our study showed a mean MBI-PA score of 39.7 (SD 5.6), which was a little higher (= healthier due to the inverse scale) than the mean MBI-PA score reported. Accordingly, only 14.6% of our participants scored high in this last subdimension of the MBI, whereas 32% of their older colleagues from the EGPRN study had a high MBI-PA score. The EGPRN study further showed that 65% of European GPs have at least one high score for burnout and 12% had three high scores. In our study only *n* = 5 (2.5%) of the GP trainees scored high in all three dimensions of the MBI, whereas *n* = 96 (47.1%) participants reached no high score at all.

### Association of sociodemographic characteristics and mental health

Linear regression models revealed that being a female GP trainee led to a higher PHQ-9 sum score (*p* < .05), which goes in line with depression statistics showing a gender gap with women being almost twice as likely as men to develop depression during their lifetime [30, 31]. There was no association between sociodemographic characteristics and PSQ-20 sum score, MBI-EE or MBI-PA. Higher age was associated with less depersonalisation in the MBI (p < .05) – another well-known phenomenon that was first described by Maslach and Jackson in 1981 in their MBI validation study with participants from human service occupations [32]. A second regression analysis (without the variable “rotation”) also revealed a negative association between age and MBI-EE. According to the well-known demands-control model by Karasek [33], job control is expected to moderate the relationship between job demands and psychological strain, which could be a possible explanation for these observations, assuming that the higher biological age is accompanied by more work experience, which in turn leads to more control over the work environment. However, a higher age does not necessarily go hand in hand with longer professional experience and it is just as conceivable that the greater life experience (with its greater arsenal of coping strategies) is responsible for this association.

### Final thoughts

It becomes very obvious that GP trainees in Germany are more burdened in terms of depression, perceived stress and possibly even burnout than the general population. Alarmingly, they seem to be almost as burdened as fully-trained GPs or hospital doctors of other disciplines, although they are usually protected from the pressures of a fully qualified GP workload. Our results are comparable to a study from Galam et al. (2013), who studied burnout in French GP trainees and demonstrated that it was frequent. We are not aware of any comparable studies from Germany.

Although this descriptive study cannot answer the question of the causes of this strain, it should be mentioned that GP trainees suffer from those stressors specific to their level of training (e.g. being held accountable for their clinical decisions for the very first time, fear of showing imperfection and new level of personal involvement) as well as from those stressors, which typically occur in general practice regardless of the level of work experience (e.g. closeness between GPs and their patients with many challenging situations, feelings of isolation, bureaucratic demands and time pressures) [2].

But what are the implications of the finding that GP trainees are almost as burdened as fully-trained GPs? We believe that the most important things are to be attentive, to train GP trainees in self-perception and the ability to talk about own problems and to increase the recognition in health policies. Before specific (political) measures such as regulations on obligatory adherence to working hours or a reduction in total working hours can be taken, we must improve our understanding of the exact causes of stress in GP trainees. However, there will only be increased efforts to reach this target group if a “cultural change” takes place at the same time, i.e. if the majority of GP educators also recognise that it would be desirable to protect and maintain the psychosocial health of GP trainees in the best possible way. We know that perfectionism which is typical for physicians (not wanting to make mistakes, not showing weakness, not revealing a knowledge gap, etc.), can also contribute to the development of stress and burnout [34], it could for example be an important learning goal of the GP training that true perfection is not possible and that mistakes are part of learning. Professional mentoring as a compulsory part of GP training could make a decisive contribution to the early detection of psychosocial stress in GP trainees, and interventions to promote their physical and mental health are necessary to ensure healthy GPs in the future [2].

### Strengths and limitations

We understand this study as a thorough cross-sectional analysis of the psychosocial burden of GP trainees. Our results might therefore be highly useful to all readers enrolled in GP training programmes and those who train young GPs in another setting. However, it is important to be aware of the predictive limitations of all cross-sectional studies: Without future longitudinal data, it is not possible to get a real idea where the detected stress burden comes from, i.e. to establish a true cause and effect relationship. Secondly, the response rate was exceptionally high and quality of data is good. All data was generated from only one GP training programme within one region of Germany and therefore should be handled with care if translated into another context, as prevalence of stress or psychosocial morbidity may vary by country of training.

## Conclusions

The results of our study suggest that GP trainees are at considerable risk to suffer from stress and even depression or burnout. Therefore, GP trainees should be trained in self-care and supported by early preventive measures as well as collegial advice (e.g. structured mentoring). Follow-up studies with larger samples could allow extended subgroup analyses to reveal further risk factors. Additionally, longitudinal prospective and qualitative studies are needed to further explore the nature and course of GP-trainees’ stress burden as well as the effectiveness of different measures promoting physician’s health.

## Data Availability

The datasets used and/or analysed during the current study are available from the corresponding author on reasonable request.

## Abbreviations

GP: General practice
GPs: General practitioner
IQR: Interquartil range
KWBW: (GERMAN) = Competence centre postgraduate (medical) education Baden-Württemberg)
KWBW Verbundweiterbildung^*plus*^*©*: Name of the training programme (www.kwbw.de) registered mark, Munich (302,018 017169)
MBI-DP: MBI-dimension: “Depersonalisation”
MBI-EE: MBI-dimension: “Emotional Exhaustion”
MBI-GS: Maslach Burnout Inventory General Survey
MBI-HSS: Maslach Burnout Inventory Human Services Survey
MBI-PA: MBI-dimension: “Personal Accomplishment”
Md: Median
PHQ-9: Patient Health Questionnaire-9
PSQ: Perceived Stress Questionnaire
PSQ-20: Perceived Stress Questionnaire-20 (short version of PSQ)
Q: Quartil
SD: Standard deviation
